# A new B-Raf inhibitor combo for advanced melanoma

**DOI:** 10.18632/oncotarget.26171

**Published:** 2018-10-02

**Authors:** Marjam-Jeanette Barysch, Joanna Mangana, Reinhard Dummer

**Affiliations:** Reinhard Dummer: Department of Dermatology, University Hospital Zurich, Switzerland; University of Zurich, Zurich, Switzerland

**Keywords:** melanoma, BRAF/MEK inhibition, encorafenib, binimetinib, COLUMBUS

During the last decade, several new treatment modalities have been developed, that impressively improved survival and quality of life in patients with advanced melanoma, who did not face any progress since more than 50 years [1]. These revolutionary developments are incomparable with other cancer types and additional progress is anticipated. In this continuous breakthrough, combination treatments have shown to facilitate superior treatment responses compared to monotherapies. The combination of checkpoint-inhibitors (CI) consisting of CTLA4 blockade with ipilimumab plus PD1 blockade with nivolumab leads to a significant higher overall-response of 58% with 19% of patients achieving complete responses [2]. In the targeted therapy field, combination of tyrosine kinase inhibitors (TKI) consisting of BRAF- plus MEK inhibitor (BRAFi and MEKi) (dabrafenib/trametinib or vemurafenib/cobimetinib) led to improved overall (OS) and progression-free survival (PFS) [3, 4]. Interestingly, the addition of MEKi ameliorates tolerability and reduces the occurrence of on -target BRAFi-related cutaneous toxicities driven by the paradoxical pathway activation. However, approximately 40%–50% of melanoma patients treated first line with the above modern treatment combinations, either fail to elicit an anti-tumor response (primary resistance) or will eventually progress at 12 months (secondary resistance); hence, further strategies are mandatory.Encorafenib is an APT-competitive BRAFi, showing a similar IC50 (half-maximal inhibitory concentration) for wild-type BRAF, V600E mutant and CRAF in cell-free biochemical assays, which might be relevant for reducing the paradoxical activation of MAPK pathway. It presents a longer dissociation half-life than dabrafenib and vemurafenib. In addition to its high potency and affinity, encorafenib is also a highly specific inhibitor of BRAF [5]. The efficacy of encorafenib in combination with the ATP-non-competitive MEK inhibitor binimetinib was investigated in the phase III clinical trial (COLUMBUS) and presented excellent efficacy in comparison to other targeted treatment combinations and yet was well tolerated. Patients were randomized 1:1:1 to COMBO450 (encorafenib 450mg+binimetinib 45mg) (*n* = 192), ENCO300 (encorafenib 300mg) (*n* = 194), or VEM (vemurafenib) (*n* = 191). At a median follow up of 16.6 months, the combination therapy led to a significant improvement in OS and in disease recurrence with a median PFS of 14.9 months compared to 7.3 months in the vemurafenib group (hazard ratio 0.54) [6].

At median follow up of 21.5 months, median OS was reported as 33.6 months (95% CI, 24.4-39.2) with COMBO450, 23.5 months (95% CI, 19.6-33.6) with ENCO300, and 16.9 months (95% CI, 14.0-24.5) with vemurafenib. The combination therapy with encorafenib plus binimetinib was better tolerated with fewer grade 3 or 4 adverse events (AEs) and a lower rate of drug discontinuation compared to ENCO300 and VEM. Lower rates of other class specific side effects such as pyrexia and photosensitivity were also observed, highlightening its’ excellent tolerability. Interestingly, and despite the numerical differences in the OS and PFS between COMBO450, vemurafenib/combimetinib in the co-BRIM and dabrafenib/trametinib in the Combi-D, the reported HR for OS was similar (0.61 versus 0.76 versus 0.68 respectively) [6, 7].

Based on the above findings, FDA approved the combination of encorafenib and binimetinib for the treatment of metastatic melanoma harboring the BRAF mutation, adding another drug combination in the pipeline. Whether it will substitute the current BRAF/MEKi options or be restricted for patients, experiencing pyrexia or photosensitivity under the already existing BRAF/MEKi combinations remains open. However, in order to stay competitive, further trials are requisite. Particularly combination therapy with checkpoint inhibitors are required, regarding multiple ongoing studies investigating combination treatments with rival BRAF/MEK-inhibitors and checkpoint inhibitors - either simultaneously or in sequence (NCT02130466, NCT02967692, NCT02224781 and NCT02908672). Furthermore, comparative prospective trials exploring the efficacy on brain metastases are needed as combined checkpoint inhibition or targeted therapy has shown excellent response in this patients collective already [8, 9].
